# Temporal validation of metabolic nodal response of esophageal cancer to neoadjuvant chemotherapy as an independent predictor of unresectable disease, survival, and recurrence

**DOI:** 10.1007/s00330-019-06310-9

**Published:** 2019-07-05

**Authors:** John M. Findlay, Edward Dickson, Cristina Fiorani, Kevin M. Bradley, Somnath Mukherjee, Richard S. Gillies, Nicholas D. Maynard, Mark R. Middleton

**Affiliations:** 1 Oxford Oesophagogastric Centre, Churchill Hospital, Oxford, OX3 7LE UK; 2 Department of Oncology, Old Road Campus Research Building, University of Oxford, Oxford, OX3 7DQ UK; 3 Department of Nuclear Medicine, Churchill Hospital, Oxford, OX3 7LE UK

**Keywords:** Esophagectomy, Prognosis, Recurrence, PET-CT, Metastases

## Abstract

**Objectives:**

We recently described metabolic nodal stage (mN) and response (mNR) of cancer of the esophagus and gastro-esophageal junction (GEJ) to neoadjuvant chemotherapy (NAC) using ^18^F-FDG PET-CT as new markers of disease progression, recurrence, and death. We aimed to validate our findings.

**Methods:**

Our validation cohort comprised all patients consecutive to our discovery cohort, staged before and after NAC using PET-CT from 2014 to 2017. Multivariate binary logistic and Cox regression were performed.

**Results:**

Fifty-one of the 200 patients had FDG-avid nodes after NAC (25.5%; i.e., lack of complete mNR), and were more likely to progress during NAC to incurable disease on PET-CT or at surgery: odds ratio 3.84 (1.46–10.1; *p* = 0.006). In 176 patients undergoing successful resection, patients without complete mNR had a worse prognosis: disease-free survival hazard ratio 2.46 (1.34–4.50); *p* = 0.004. These associations were independent of primary tumor metabolic, pathological response, and stage. In a hybrid pathological/metabolic nodal stage, avid nodal metastases conferred a worse prognosis than non-avid metastases. Lack of complete mNR predicted recurrence or death at 1 and 2 years: positive predictive values 44.4% (31.7–57.8) and 74.1% (56.6–86.3) respectively.

**Conclusions:**

This study provides temporal validation for mNR as a new and independent predictive and prognostic marker of esophageal and GEJ cancer treated with NAC and surgery, although external validation is required to assess generalizability. mNR may provide surrogate information regarding the phenotype of metastatic cancer clones beyond the mere presence of nodal metastases, and might be used to better inform patients, risk stratify, and personalize management, including adjuvant therapy.

**Key Points:**

*• We previously described metabolic nodal response (mNR) of esophageal cancer to neoadjuvant chemotherapy using*^*18*^*F-FDG PET-CT as a predictor of unresectable disease, early recurrence, and death.*

*• We report the first validation of these findings. In an immediately consecutive cohort, we found consistent proportions of patients with and without mNR, and associations with abandoned resection, early recurrence, and death.*

*• This supports mNR as a new and actionable biomarker in esophageal cancer. Although external validation is required, mNR may provide surrogate information about the chemosensitivity of metastatic subclones, and the means to predict treatment success, guide personalized therapy, and follow-up.*

**Electronic supplementary material:**

The online version of this article (10.1007/s00330-019-06310-9) contains supplementary material, which is available to authorized users.

## Introduction

We recently reported the new concepts of metabolic nodal stage (mN) and response (mNR) of esophageal and gastro-esophageal junction (GEJ) cancer to neoadjuvant chemotherapy (NAC) [1, 2]. We found that patients with FDG-avid nodes within a standard lymphadenectomy field on ^18^F-fluorodeoxyglucose (FDG) positron emission tomography-computed tomography (PET-CT) that persisted despite NAC (i.e. lacking a complete metabolic nodal response, CMR) were more likely to suffer disease progression during NAC which prevented resection (i.e., distant metastases, or locally unresectable disease). Furthermore, if resection was performed successfully, these patients were more likely to suffer disease recurrence. This was independent of pathological stage, and both pathological and metabolic regression of the primary tumor [1, 2], suggesting mNR to be a surrogate of regression of nodal metastases to NAC. Indeed, in some patients notable disparity was seen between primary and nodal response, suggesting mNR might represent a surrogate of the chemosensitivity of different clones, and perhaps by extrapolation occult distant metastatic clones.

mNR therefore represents a potentially novel and actionable biomarker of the success both of NAC and radical lymphadenectomy, for which there is an urgent need [3–6]. Such a marker might have the potential to monitor response to NAC and perhaps personalize therapy, while also better prognosticating before surgery, and stratifying the risk of recurrence thereafter. To our knowledge, no studies have subsequently assessed mNR as a marker of disease progression and recurrence. We aimed to perform the first validation study of mNR as a marker of disease progression and recurrence, using a temporal cohort of patients immediately consecutive to our discovery cohort.

## Methods

### Study design and approval

We routinely stage and restage patients with esophageal or GEJ cancer using ^18^F-FDG PET-CT, and retrospectively identified all such patients receiving NAC from January 2014 to June 2017. The study was approved by the Oxford University Hospitals NHS Foundation Trust Research and Development committee.

### Inclusion and exclusion criteria

We included all patients with histologically confirmed cancer of the esophagus or GEJ, staged in our institution with ^18^F-FDG PET-CT, undergoing NAC before being restaged with ^18^F-FDG PET-CT. We excluded patients treated with neoadjuvant chemoradiotherapy, as well as those undergoing PET-CT in other centers.

### Staging and restaging

All examinations were reported by subspecialist consultant gastrointestinal radiologists and reviewed at a specialist esophagogastric cancer multidisciplinary team (MDT) meeting, using the TNM 7th edition [7]. Patients underwent intravenous and oral contrast-enhanced CT chest abdomen and pelvis, as previously reported [1, 2]. In the absence of unequivocal incurable disease, patients then underwent PET-CT (see below), followed by laparoscopy (without peritoneal cytology) for tumors extending below the diaphragm. Endoscopic ultrasound (EUS) was performed selectively. Examinations were reported. Patients were restaged 4–6 weeks after NAC.

PET-CT was performed from January 2014 using a General Electric (GE) Discovery 690 64-slice system, then also a GE Discovery 710 from September 2014 (both 90 min post 4 MBq/Kg FDG), all using ordered subset expectation maximization (OSEM) reconstruction; after 1 November 2014, a Bayesian penalized likelihood (BPL) reconstruction technique was used (Q.Clear) without intravenous contrast. Any staging studies initially reconstructed with OSEM and undergoing a subsequent restaging examination with BPL had their baseline studies reconstructed with BPL to ensure validity. Examinations were independently reported by two dedicated PET-CT radiologists, dual trained in nuclear medicine and clinical radiology.

### Data

PET-CT data comprised primary tumor maximum standardized uptake value (SUVmax), the presence and number of FDG-avid nodes (mN, as previously defined: mN1 1–2 avid nodes, mN2 > 2), in both cases, a target SUVmax greater than mediastinal blood pool being avid [1]; restaging SUVmax (used to calculate metabolic tumor response [mTR], % reduction SUVmax normalized for background SUVmax [2.5], and PERCIST classification [8]); metastatic disease; mNR as previously defined (complete metabolic response [CMR] to background mediastinal blood pool avidity, partial metabolic response [PMR; reduction in mN stage or % reduction in nodal SUVmax > 30%], stable metabolic disease [SMD; no change in mN stage, or nodal SUVmax < 30%], and progressive metabolic disease [PMD; progression in mN stage, or increase nodal SUVmax > 30%]).

Pathological data comprised pathological stage [7], and primary tumor regression (Mandard tumor regression grade 1–3 being response, 4–5 no response [2, 9]). Survival data comprised recurrence and death (censored on 23 March 2018). Disease progression to incurable disease during NAC was defined as either metastatic disease on imaging (confirmed by MDT discussion ± confirmatory imaging/histopathology) or unresectable disease at surgery (confirmed intra-operatively by frozen section histopathology, or consensus of two consultant surgeons).

### Neoadjuvant chemotherapy

Patients received a variety of regimens based on MDT consensus and trial enrolment: either 2 cycles of doublet therapy (cisplatin/oxaliplatin and 5-fluorouracil/capecitabine) or 3 or 4 cycles of pre- ± post-operative triplet therapy (epirubicin, cisplatin/oxaliplatin and 5-fluorouracil/capecitabine) as previously described [10–14].

### Surgery

Esophagectomy (for esophageal or GEJ type I–II cancers) or extended total gastrectomy (types II–III) was performed typically 2 weeks after restaging, via left thoracoabdominal, Ivor-Lewis (right thoracotomy plus laparotomy/laparoscopy), and three-stage approaches (laparotomy/laparoscopy, thoracotomy plus neck dissection). A minimum two-field lymphadenectomy was performed; for mid/distal esophageal/type 1 GEJ tumors, this included subcarinal, aortopulmonary window, and left gastric nodes, and for distal/GEJ tumors, left gastric and common hepatic artery nodes.

### Follow-up

Patients were reviewed clinically at 2 and 6 weeks after surgery, 3 months to 1 year and 6–12 months thereafter for at least 5 years. Investigations for recurrence were performed only on the basis of clinical suspicion, typically CT or PET-CT for extra-luminal recurrence, and endoscopy for luminal.

### Statistical analysis

Analysis was performed using R v3.0.2 [15]. Groups were compared using Fisher’s exact test. Univariate binary logistic and Cox regression analysis were performed using all individual data fields. Multivariate binary logistic and Cox regression were performed using variables with *p* < 0.1 on univariate analysis for inclusion in the final multivariate model. The sole patient with an adenosquamous carcinoma was excluded from univariate analysis of cell type, being a perfect separator. The final multivariate binary logistic regression models for disease progression comprised mTR and restaging mN; for unresectable disease at surgery, restaging mN and pre-treatment grade of differentiation; and for disease-progression overall, restaging mN and clinical N stage. The final multivariate Cox models for survival comprised mNR, ypT, ypN, ypV (except for overall survival, as *p* > 0.1 on univariate analysis), ypL, resection margin status, pTR, and mTR. For the model assessing a pNmN hybrid, the models were as above, having excluded mNR and ypN due to multicollinearity. The final multivariate binary logistic regression model for early recurrence/death comprised of mnR, ypT, ypN, ypL, resection margin status, pTR, and mTR. Similarly, the model assessing a pNmN hybrid excluded mNR and ypN. Survival metrics were calculated using the Kaplan-Meier method, and follow-up using the reverse Kaplan-Meier method, excluding patients dying in hospital due to post-operative complications. Kaplan-Meier curves were generated using survfit (Survival Package v2.42-3) using the log-rank test.

## Results

### mN stage and response

Two hundred patients were included (Table 1). Eighty-one (40.5%) had avid nodes before NAC. Thirty-eight (46.9%) underwent CMR. Eight (4.00% overall) previously mN0 patients developed avid nodes. In total, 51 patients (25.5%) had avid nodes following NAC. This was consistent with the development cohort: 64/280 (22.9%; *p* = 0.617). PET reconstruction algorithms did not influence mNR, mTR, and detection of metastatic disease (Supplementary Table 1).

**Table 1:** Patients

Characteristic	All patients (*n* = 200)	Survival analysis (*n* = 176)
Age (median, IQR)	70.0 (63.0–74.0)	70.0 (63.0–74.0)
Gender		
Male	159 (79.5%)	137 (77.8%)
Female	41 (20.5%)	39 (22.2%)
Tumor cell type		
AC	183 (91.5%)	161 (91.5%)
SCC	16 (8.00%)	14 (7.95%)
AS	1 (0.50%)	1 (0.58.%)
Grade of differentiation		
Well	4 (2.00%)	3 (1.70%)
Moderate	93 (46.5%)	86 (48.9%)
Poor/undifferentiated	103 (51.5%)	87 (49.4%)
Tumor site		
Mid esophageal	15 (7.50%)	12 (6.82%)
Distal esophageal	77 (38.5%)	67 (38.1%)
GEJ1	55 (27.5%)	53 (29.5%)
GEJ2	29 (14.5%)	25 (14.2%)
GEJ3	24 (12.0%)	19 (10.8%)
Pre-chemotherapy mN stage		
0	119 (59.5%)	108 (61.4%)
1	47 (23.5%)	40 (22.7%)
2	34 (17.0%)	28 (15.9%)
Metabolic nodal response (mNR)		
No avid nodes	111 (55.5%)	102 (58.0%)
CMR	38 (19.0%)	35 (19.9%)
PMR	9 (4.5%)	8 (4.54%)
SMD	25 (12.5%)	20 (11.4%)
PMD	9 (4.5%)	6 (3.41%)
de novo avid nodes	8 (4.00%)	5 (2.84%)
mTR		
% Reduction SUVmax (median, IQR)	42.4% (12.8–60.6%)	45.5% (15.8–62.9%)
CMR	15 (7.50%)	15 (8.53%)
T stage (clinical or pathological)	Clinical T stage	Pathological T stage, ypT
1b	5 (2.50%)	29 (16.5%)
2	34 (17.0%)	20 (11.4%)
3	133 (66.5%)	116 (65.9%)
4	28 (14.0%)	11 (6.25%)
N stage (clinical or pathological)	Clinical N stage	Pathological N stage, ypN
0	64 (32.0%)	67 (38.1%)
1	83 (41.5%)	43 (24.4%)
2	47 (23.5%)	38 (21.6%)
3	6 (3.00%)	28 (15.9%)
ypV	NA	
0		102 (58.0%)
1		73 (41.5%)
Not known		1
ypL	NA	
0		101 (57.4%)
1		74 (42.0%)
Not known		1
Resection margin status		
Clear		147 (83.5%)
Involved		29 (16.5%)
pTR		
No response		137 (49.6%)
Response		38 (21.6%)
Not known		1

*AC*, adenocarcinoma; *SCC*, squamous cell carcinoma; *AS*, adenosquamous carcinoma (excluded from multivariate analysis as perfect separator); *IQR*, interquartile range; *mTR*, metabolic tumor response; *CMR*, complete metabolic response; *PMR*, partial metabolic response; *SMD*, stable metabolic disease; *PMD*, progressive metabolic disease; *ypT*, pathological tumor stage after chemotherapy; *ypN*, pathological nodal stage; *ypV*, pathological venous invasion; *ypL*, pathological lymphatic invasion; *pTR*, pathological tumor response

### Progression to incurable disease on restaging PET-CT

Incurable disease was identified in 10 (5.00%) patients. No pre-treatment factors were associated with this. On multivariate binary logistic regression, both the presence of avid nodes at restaging (mN stage 2) and lack of mTR (% reduction in primary tumor SUVmax) were associated with coexistent metastatic disease (Table 2). Five of 51 (9.80%) patients with avid nodes after NAC had metastatic disease, compared with 5 of 149 (3.36%) without (*p* = 0.127).

**Table 2:** Disease progression: multivariate binary logistic regression

Characteristic	Odds ratio (OR; 95% confidence interval)	*p*
Progression to overt metastatic disease on PET-CT		
Restaging mN		
mN0	Ref	
mN1	0.78 (0.08–7.38)	0.832
mN2	4.37 (1.03–18.6)	0.045
mTR		
% reduction in SUVmax	0.11 (0.02–0.56)	0.008
Progression to unsuspected unresectable disease at surgery		
Restaging mN		
mN0	Ref	
mN1	6.25 (1.24–31.6)	0.027
mN2	8.28 (1.59–43.2)	0.012
Grade		
Well/moderate	Ref	
Poor	5.85 (1.14–30.0)	0.034
FDG-avid nodes after NAC		
No	Ref	
Yes	7.67 (1.94–30.4)	0.004
Grade		
Well/moderate	Ref	
Poor	0.17 (0.04–0.91)	0.038
Disease progression overall		
Restaging mN		
mN0	Ref	
mN1	2.79 (0.78–10.0)	0.114
mN2	4.62 (1.48–14.4)	0.001
Staging N		
0	Ref	Ref
1+	3.43 (0.0.73–16.1)	0.118
FDG-avid nodes after NAC		
No	Ref	
Yes	3.84 (1.46–10.1)	0.006
Staging N		
0	Ref	
1+	3.79 (0.83–17.2)	0.085

*mN*, metabolic nodal stage; *NAC*, neoadjuvant chemotherapy; *mTR*, metabolic tumor response; *FDG*, fluorodeoxyglucose

### Progression to unresectable disease at surgery

A further 2 patients without metastases evident on restaging PET-CT did not proceed to surgery for medical reasons (both had avid nodes). One hundred eighty-eight proceeded to surgery. Unsuspected unresectable disease was encountered in 10 (5.32%). On multivariate analysis, this was associated with the presence of avid nodes following NAC: OR 7.67 (1.94–30.4; *p* = 0.004; Table 2).

### Disease progression overall

Overall, avid nodes following NAC predicted disease progression: OR 3.84 (1.46–10.1; *p* = 0.006), independent of all variables including clinical N stage. As with progression on PET-CT, this association was primarily seen for patients with mN2 disease after NAC.

### Predicting unresectable disease at surgery

Before surgery, 44 of 188 patients (23.4%) had avid nodes following NAC. Of these, 6 (14.0%) had an abandoned resection, compared with 4 of 144 without (2.78%; *p* = 0.011; 6 true positives, 38 false positives, 4 false negatives, and 140 true negatives) The presence of avid nodes despite NAC was 60.0% sensitive (26.2–87.8) and 78.7% specific (71.9–84.4) for predicting unsuspected unresectable disease. Positive predictive value (PPV) was 13.6% (8.13–22.0), and negative predictive value (NPV) was 97.2% (94.2–98.9%).

These proportions were less than the development cohort, in which 11 of 43 (25.6%) patients with avid nodes had an abandoned resection, compared with 15 of 197 (7.61%) without. Rates of abandoned resections halved from 10.5 to 5.32%.

### Prognosis

Two patients (1.06%) died in hospital of post-operative complications and were excluded. Of the remaining 176 patients (Table 1), 55 died (31.3%) and 59 (33.5%) developed recurrence during follow-up. Median OS, DFS, and recurrence were not reached (respective 95% CI 1067 days—not reached; 770—not reached; 872—not reached). Median follow-up was 783 days (737–886).

**Fig. 1:**
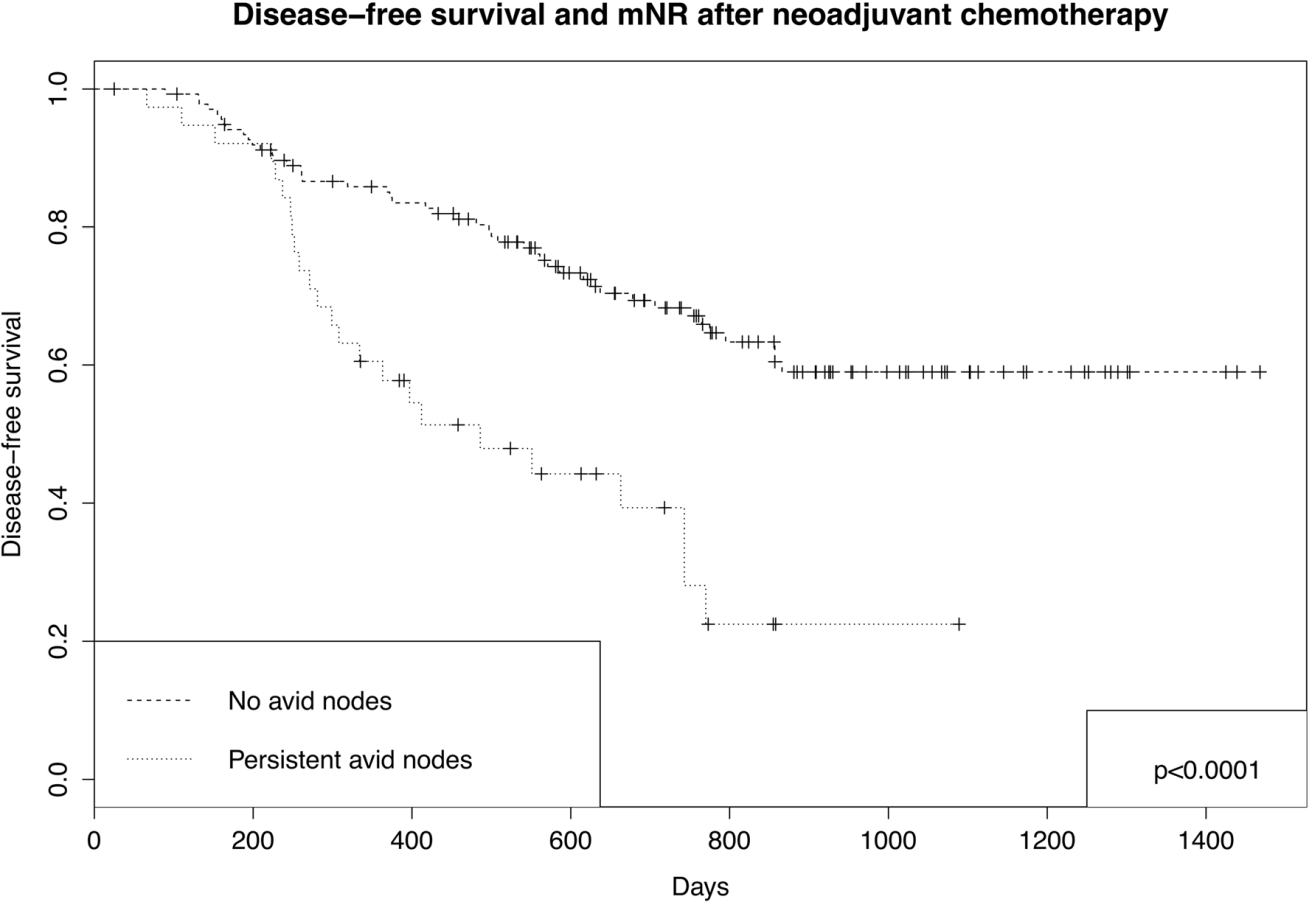
Disease-free survival and mNR after neoadjuvant chemotherapy; *p* values generated using log-rank test

On multivariate Cox analysis, the presence of avid nodes following NAC was associated with death and recurrence following resection: OS HR 2.46 (1.34–4.51; *p* = 0.004), DFS HR 1.90 (1.10–3.31; *p* = 0.022), and time to recurrence HR 2.02 (1.11–3.66; *p* = 0.021; Table 3; Fig. 1). This was independent of similar negative associations with progressive ypN stage. No other factors, including mTR (either reduction in SUVmax, or PERCIST), were independently associated with prognosis. There were no clinically or statistically significant differences when mNR was quantified using mN stage after NAC (i.e., the number of avid nodes). These HR were similar to the development cohort: OS 1.75 (0.99–3.07; *p* = 0.053), DFS 2.03 (1.16–3.55; *p* = 0.013), and recurrence 2.06 (1.10–3.83; *p* = 0.023).

**Table 3:** Prognosis: multivariate Cox regression analysis

Characteristic	Hazard ratio (HR; 95% confidence interval); *p* value		
	Overall survival	Disease-free survival	Time to recurrence
mNR			
No avid nodes/CMR	Reference	Reference	Reference
PMR/SMD/PMD	2.46 (1.34–4.51; 0.004)	1.90 (1.10–3.31; 0.022)	2.02 (1.11–3.66; 0.021)
ypT stage			
1b	Reference	Reference	Reference
2	0.51 (0.09–2.91; 0.449)	0.83 (0.21–3.21; 0.784)	0.96 (0.23–4.04; 0.953)
3	0.75 (0.22–2.61; 0.656)	0.83 (0.28–2.46; 0.739)	0.80 (0.24–2.66; 0.720)
4	1.45 (0.34–6.49; 0.594)	1.69 (0.46–5.22; 0.429)	1.62 (0.39–6.38; 0.509)
ypN stage			
0	Reference	Reference	Reference
1	2.19 (0.81–5.95; 0123)	3.04 (1.26–7.32; 0.013)	3.19 (1.19–8.54; 0.021)
2	2.88 (1.07–7.74; 0.036)	3.78 (1.54–9.26; 0.004)	4.45 (1.66–12.1; 0.003)
3	3.81 (1.34–10.8; 0.012)	4.62 (1.81–11.8; 0.001)	4.21 (1.47–12.1; 0.007)
ypV stage			
0	Not included as univariate *p* > 0.1	Reference	Reference
1		0.90 (0.50–1.61; 0.715)	0.83 (0.44–1.57; 0.572)
ypL stage			
0	Reference	Reference	Reference
1	1.34 (059–3.06; 0.488)	1.54 (0.72–3.29; 0.264)	1.80 (0.78–4.15; 0.167)
Resection margin			
Clear	Reference	Reference	Reference
Involved	2.00 (0.97–4.13; 0.062)	1.79 (0.95–3.38; 0.073)	1.43 (0.69–2.93; 0.333)
pTR			
No response	Reference	Reference	Reference
Response	0.69 (0.19–1.90; 0.390)	0.79 (0.32–1.94; 0.610)	0.83 (0.32–2.21; 0.715)
mTR			
%Reduction SUVmax	0.79 (0.33–1.86; 0.584)	0.88 (0.40–1.92; 0.753	0.77 (0.34–1.76; 0.536)
pN Mn hybrid, adjusted for variables above other than pN and mNR			
pN mN hybrid			
pN0 mN0	Reference	Reference	Reference
pN– mN+	1.91 (0.23–15.6; 0.546)	4.00 (0.83–19.4; 0.085)	5.60 (1.08–29.1; 0.040)
pN+ mN –	3.87 (1.68–8.89; 0.001)	5.75 (2.75–12.9; < 0.001)	6.86 (2.68–17.6; < 0.001)
pN+ mN+	10.5 (4.37–25.2; < 0.001)	11.1 (4.68–26.1; < 0.001)	13.5 (5.00–36.5; < 0.001)

*CMR*, complete metabolic response; *PMR*, partial metabolic response; *SMD*, stable metabolic disease; *PMD*, progressive metabolic disease; *mNR*, metabolic nodal response; *mTR*, metabolic tumor response; *ypT*, pathological tumor stage after NAC; *ypN*, pathological nodal stage; *ypV*, pathological venous stage; *ypL*, pathological venous stage; *pTR*, pathological tumor response

### Hybrid pathological metabolic nodal stage

As both mNR and ypN independently predicted prognosis, we considered a composite. Overall 109 patients had nodal metastases (ypN+), while 38 had avid nodes (mN+). Considering post-NAC mN, a predictor of ypN, there were 31 true positives, 7 false positives, 78 false negatives, and 60 true negatives. mNR was 28.4% sensitive (20.2–37.9), and 89.5% specific (79.7–95.7), PPV 81.6% (76.4–90.5), and NPV 43.5% (40.0–47.0).

**Fig. 2:**
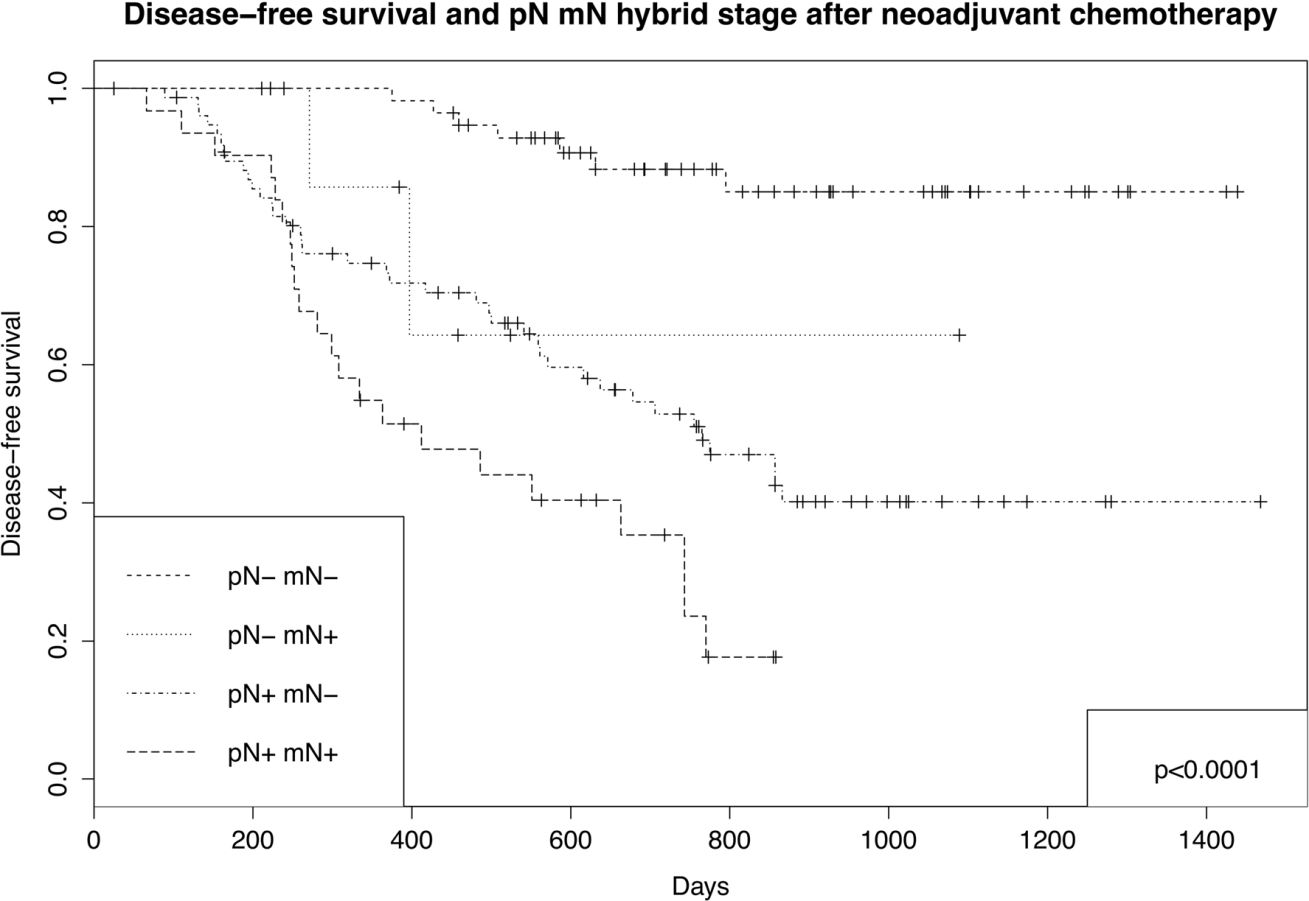
Disease-free survival pN mN hybrid stage after neoadjuvant chemotherapy; *p* values generated using log-rank test

This ypNmN stage was associated with prognosis (Table 3; Fig. 2): relative to pN0mN0 disease, patients with non-avid nodal metastases (ypN + mN−) had a worse prognosis. However, prognosis was even worse for patients with avid nodal metastases (ypN + mN+). Interestingly, the small number with ypN-mN+ disease (i.e., presumed false avid positive) demonstrated a trend towards worse prognosis for DFS (*p* = 0.085) with significantly worse recurrence, suggesting the possibility of pathologically missed micro-metastases.

### Predicting early recurrence and death

One hundred twenty-nine of 161 (78.2%) patients were alive and disease-free at 1 year, reducing to 68 of 128 (53.1%) at 2 years. On multivariate regression, lack of complete mNR was associated with recurrence and death at both time points: OR 4.18 (1.56–11.2; *p* = 0.004) and 3.63 (1.26–10.5; *p* = 0.017) respectively. This was again independent of associations with ypN (Table 4). The associations with ypN were primarily for recurrence/death within 1 year, rather than 2 years. At 2 years, recurrence and death were associated with hybrid pNmN stage.

**Table 4:** Predicting early recurrence and death: multivariate binary logistic regression

Characteristic	Odds ratio (OR; 95% confidence interval; *p* value)	
	Within 1 year	Within 2 years
mNR		
No avid nodes/CMR	Reference	Reference
PMR/SMD/PMD	4.18 (1.56–11.2; 0.004)	3.63 (1.26–10.5; 0.017)
ypT stage		
1b	Reference	Reference
2	0.50 (0.45–4.97; 0.556)	0.82 (0.11–6.01; 0.843)
3	0.30 (0.05–2.07; 0.217	1.23 (0.27–5.50; 0.789)
4	0.47 (0.05–4.54; 0.514)	5.18 (0.58–46.5; 0.142)
ypN stage		
0	Reference	Reference
1	18.5 (1.89–182; 0.012)	2.24 (0.65–7.77; 0.204)
2	49.7 (5.01–493; < 0.001)	2.93 (0.81–10.6; 0.101)
3	74.3 (6.66–829; < 0.001)	4.79 (1.14–20.1; 0.032)
ypL stage		
0	Reference	Reference
1	0.68 (0.190–2.43; 0.556)	1.54 (0.72–3.29; 0.332)
Resection margin		
Clear	Reference	Reference
Involved	1.59 (0.54–4.71; 0.399)	2.91 (0.79–10.7; 0.108)
pTR		
No response	Reference	Reference
Response	0.47 (0.09–2.55; 0.383)	1.36 (0.38–4.91; 0.637)
mTR		
%Reduction SUVmax	1.06 (0.26–4.36; 0.993)	1.06 (0.28–4.09; 0.931)
pN mN hybrid adjusted for all variables above, other than ypN and mNR		
pN mN hybrid		
pN0 mN0	Reference	Reference
pN − mN+	NA	11.4 (0.85–152; 0.067)
pN + mN−	NA	3.47 (1.07–11.3; 0.039)
pN + mN+	NA	9.19 (2.15–39.3; 0.003)

*CMR*, complete metabolic response; *PMR*, partial metabolic response; *SMD*, stable metabolic disease; *PMD*, progressive metabolic disease; *mNR*, metabolic nodal response; *mTR*, metabolic tumor response; *ypT*, pathological tumor stage; *ypL*, pathological venous stage; *pTR*, pathological tumor response

At 1 year, 16 of 36 (44.4%) patients with persistent avid nodes had either died or developed recurrence, compared with 20 of 129 (15.5%) without (*p* < 0.001 Fisher’s exact test). Lack of a nodal CMR was 44.4% sensitive (27.9–61.9) and 84.5% specific (77.1–90.3), with PPV 44.4% (31.7–57.8) and NPV 84.5 (80.1–88.1).

At 2 years, 20 of 27 (74.1%) patients with persistent avid nodes had either died or developed recurrence, compared with 38 of 99 without (38.4%; *p* = 0.001). Lack of nodal CMR was 34.5% sensitive (22.5–48.1) and 89.7% specific (79.9–95.8), with PPV 74.1% (56.6–86.3) and NPV 61.6% (56.7–66.3).

## Discussion

We previously reported lack of complete metabolic nodal response of esophageal cancer to NAC to be a new and independent predictor of disease progression to unresectable disease [1], death, and recurrence [2]. In this validation study of an immediately consecutive cohort of 200 patients, we confirmed these associations and were able to risk stratify recurrence and death within 1 and 2 years. Compared with our development cohort, effect sizes were entirely consistent for prognosis. That for unresectable disease while significant and consistent, was less clear, reflective of a reduction in event rate overall with time.

These findings have a number of implications. Most immediately, mNR appears to a simple way to risk stratify patients before surgery, firstly identifying patients at higher risk of occult disease resulting in an abandoned resection. Guidelines presently recommend restaging with CT to exclude disease progression before surgery [16]. We previously reported PET-CT to be more sensitive [1]; however, this still failed to identify incurable disease in 5% of our patients. Identifying lack of complete mNR might allow these patients to undergo additional investigations (such as laparoscopy, thoracoscopy, or magnetic resonance imaging). These patients may also benefit from more personalized and realistic information about their immediate risk of an abandoned resection, and medium- and long-term prognosis if surgery is successful. While this may not routinely alter practice—other than for patients unclear as to whether to proceed with surgery—patients can receive more realistic information before consenting to surgery. While there is little evidence or consensus as to follow-up, mNR might be incorporated into decision support tools for targeted surveillance, and future editions of the TNM classification. Finally, the suggestion that patients with avid nodal disease, seemingly in the absence of pathological nodal disease, have a worse prognosis than those without either is notable. Such instances might be dismissed as false positives on PET-CT, but the converse might be true, with these instances identifying occult but viable nodal micrometastases.

Biologically, this statistical independence of pN and mNR is intriguing. As evidenced by the prognostic significance of a novel pNmN hybrid stage, avid nodal metastases confer a worse prognosis than non-avid metastases. This suggests persistent nodal avidity to be a surrogate of a worse cancer phenotype. While the reasons for nodal avidity are complex and incompletely understood (perhaps reflecting mutational burden disrupting glucose utilization, in conjunction with tumor micro-environment, perfusion, and hypoxia), at a simplistic level, it may be possible to infer chemo(in)sensitivity. We previously reported primary tumor response on PET-CT (mTR) to be an imperfect surrogate of pathological response (pTR) [17], in agreement with other groups [18], including its successful use within the MUNICON trial [19]. Esophageal cancer comprises a limited number of cancer clones, which vary genomically and probably phenotypically, including their chemosensitivity and propensity to metastasize [20]. Nodal metastases may therefore comprise just one or two selected oligoclones [21, 22]. We, and other groups, have previously reported dramatic clonal evolution within the primary tumor during the selection pressure of NAC [23, 24], with chemo-resistant clones tending to persist or emerge, while chemosensitive clones are lost. Lack of mNR may therefore be an indicator of chemo-resistant clones within resectable lymph nodes, and therefore by extrapolation, a surrogate for the occult distant nodal or hematogenous micrometastases that are responsible for recurrence. This hypothesis is supported by evidence that nodal downstaging, evidenced by imaging in conjunction with pN stage, represents an independent prognostic marker, likely a surrogate of pathological nodal regression [25, 26]. Whether this plausibly might be used to guide neoadjuvant and adjuvant therapy is unknown.

While this study provides temporal validation of mNR as a biomarker, we acknowledge a number of limitations. Firstly, this represents a single-center study with a degree of subjectivity inherent to all imaging studies (although one subject to a rigorous MDT process); whether these findings can be generalized to other centers and imaging platforms is not clear. However, similar results have been reported from Japan [27] following largely platinum-based NAC as a marker of overall survival (although recurrence was not assessed). While we previously found no differences across reconstruction platforms [2], these, and other variables (such as NAC regimen) inevitable of evolving practice, may exert subtle effects we were unable to identify. We were also unable to reliably adjust for adjuvant therapy, and there may be additional factors that we have failed to identify. Secondly, detection biases have been introduced by “on-demand” investigations for recurrence rather than surveillance imaging. Thirdly, while we found consistent direction and sizes of effect for prognosis compared with our development cohort, we saw fewer patients overall with occult unresectable disease resulting in an abandoned resection. This probably reflects improvements in care with time, and hence while the association persisted, its effect size is likely to be less accurate, and requires further validation.

In conclusion, in a cohort of 200 patients immediately consecutive to our discovery cohort, we found lack of a complete mNR (i.e., the development or persistence of avid nodes despite NAC) to again be an independent and seemingly consistent marker of disease progression, recurrence, and death after surgery. This temporal validation provides supportive evidence justifying the assessment of mNR as a new candidate biomarker for these endpoints in external validation sets. If supported, mNR might represent a new mechanism by which to both risk stratify patients and personalize therapy with esophageal and GEJ cancer.

## Electronic supplementary material

Supplementary Table 1 (DOCX 15 kb)

## Abbreviations

BPL: Bayesian penalized likelihood
CI: Confidence interval
CMR: Complete metabolic response
CT: Computed tomography
DFS: Disease-free survival
EUS: Endoscopic ultrasound
FDG: ^18^F-fluorodeoxyglucose
GE: General Electric
GEJ: Gastro-esophageal junction
MDT: Multidisciplinary team
mN: Metabolic nodal stage
mNR: Metabolic nodal response
NPV: Negative predictive value
mTR: Metabolic tumor response
NAC: Neoadjuvant chemotherapy
NHS: National Health Service
OS: Overall survival
OSEM: Ordered subset expectation maximization
PERCIST: Positron Emission Tomography Response Criteria in Solid Tumors
PET-CT: Positron emission tomography-computed tomography
PMD: Progressive metabolic disease
PMR: Partial metabolic response
PPV: Positive predictive value
SMD: Stable metabolic disease
SUVmax: Maximum standardized uptake value
ypN: Pathological nodal stage after chemotherapy
ypT: Pathological tumor stage after chemotherapy
