# Supplementary material for: Temporal validation of metabolic nodal response of esophageal cancer to neoadjuvant chemotherapy as an independent predictor of unresectable disease, survival, and recurrence
Source: Eur Radiol. 2019 Jul 5;29(12):6717–27. doi: 10.1007/s00330-019-06310-9 (PMC6828837; doi:10.1007/s00330-019-06310-9)
Supplement: Supplementary file 1 — (DOCX 15 kb) [file 330_2019_6310_MOESM1_ESM.docx]

**Supplementary table 1** – PET-CT endpoints and reconstruction algorithm

| Characteristic | OSEM | BPL | p value |
| --- | --- | --- | --- |
| mNR  *No avid nodes/CMR*  *PMR/SMD/PMD* | 50 (75.8%)  16 (24.2%) | 99 (73.9%)  35 (26.1%) | 0.883^a^ |
| mTR  % Reduction SUVmax  median (IQR) | 35.2% (12.8-52.%) | 45.5% (12.9-64.4%) | 0.276^b^ |
| Metastatic disease  M0  M1 | 63 (94.5%)  3 (4.55%) | 127 (94.8%)  7 (5.22%) | 1.000^a^ |

A=Fisher’s exact test. B=Mann-Witney U test
